# Functional data analysis for identifying nonlinear models of gene regulatory networks

**DOI:** 10.1186/1471-2164-11-S4-S18

**Published:** 2010-12-02

**Authors:** Georg Summer, Theodore J Perkins

**Affiliations:** 1 Ottawa Hospital Research Institute, Ottawa, Ontario, Canada; 2 Upper Austrian University of Applied Sciences, Hagenberg, Austria; 3 Department of Biochemistry, Microbiology and Immunology, University of Ottawa, Ottawa, Ontario, Canada

## Abstract

**Background:**

A key problem in systems biology is estimating dynamical models of gene regulatory networks. Traditionally, this has been done using regression or other ad-hoc methods when the model is linear. More detailed, realistic modeling studies usually employ nonlinear dynamical models, which lead to computationally difficult parameter estimation problems. Functional data analysis methods, however, offer a means to simplify fitting by transforming the problem from one of matching modeled and observed dynamics to one of matching modeled and observed time derivatives–a regression problem, albeit a nonlinear one.

**Results:**

We formulate a functional data analysis approach for estimating the parameters of nonlinear dynamical models and evaluate this approach on data from two real systems, the gap gene system of *Drosophila* melanogaster and the synthetic IRMA network, which was created expressly as a test case for genetic network inference. We also evaluate the approach on simulated data sets generated by the GeneNetWeaver program, the basis for the annual DREAM reverse engineering challenge. We assess the accuracy with which the correct regulatory relationships within the networks are extracted, and consider alternative methods of regularization for the purpose of overfitting avoidance. We also show that the computational efficiency of the functional data analysis approach, and the decomposability of the resulting regression problem, allow us to explicitly enumerate and evaluate all possible regulator combinations for every gene. This gives deeper insight into the the relevance of different regulators or regulator combinations, and lets one check for alternative regulatory explanations.

**Conclusions:**

Functional data analysis is a powerful approach for estimating detailed nonlinear models of gene expression dynamics, allowing efficient and accurate estimation of regulatory architecture.

## Background

A key problem in systems biology is estimating dynamical models of gene regulatory networks. The mathematical modeling of expression dynamics, combined with model parameter estimation, has been crucial to unraveling complex regulatory programs [1], to recognizing the robustness of the regulatory architecture of the segment polarity genes to variations in initial conditions and parametric variation [2-4], to studying mechanisms of robustness and evolution of the control of the cell cycle in yeast [5,6], to identifying surprising shifts in the expression domains of the gap genes and the regulatory interactions responsible [7,8], and to numerous other studies (e.g., [9-16]).

Methods for estimating dynamical models depend on the form of the model and of the data available. We focus on the problem of estimating differential equation models of gene network dynamics based on time series data. Assuming one notion of expression is associated to each gene–for example, mRNA or protein expression level, but not both–then a generic ordinary differential equation (ODE) model for *N* genes can be formulated as(1)

where **x** is the vector of expression levels of the *N* genes, and **f** produces a vector of time derivatives of expression depending on the current expression levels and on some adjustable parameters *θ.* These parameters typically encode such features as kinetic rates for the production and decay of gene products, and regulatory influences between the genes. The regulatory architecture of the system–that is, which genes' expression derivatives depend on which other genes' expression–may be made explicit in the function **f** (e.g., [2]), or it may be implicit in the parameters (e.g., [7,8]), in which case optimizing the parameters implicitly determines network architecture. Extensions of our work to modeling both mRNA and protein levels of expression, for example, are straightforward, as would be extensions to functions **f** that depend on time or to delay differential equations, where the derivatives depend on the state of the system in the past. We will also assume that the expression data is collected from the wild type network, though initial conditions may vary. Knock-out or over-expression data has also proven useful in genetic network inference, both in theory [17] and in practice [18]. However, wild-type data is far more common and easier to generate than genetic perturbation data.

To introduce the dynamics estimation approach we investigate, suppose for simplicity that we have access to a single time series **y**(*t*_0_),**y**(*t*_1_),…*,***y**(*t_T_*)*,* where each vector contains possibly-noisy observed expression values for all *N* of the genes. Suppose further that we have chosen the form of our model, **f**(**x**, *θ*)*.* Most often, parameters of an ODE model are estimated by minimizing the squared error between modeled and observed expression(2)

where **x**(*t_i_*) denotes the solution to the ODE (Eq. 1) with parameters *θ.* The initial conditions are often taken from the observed data, **x**(*t*_0_) = **y**(*t*_0_)*,* or they may be part of the parameters *θ.* Even when the ODE model is linear, so that  for some matrix *A,* this optimization is not trivial. The solution to such an ODE is given by the matrix exponential **x**(*t*) = *e^At^***x**(*t*_0_), so that the dependence of the error on the parameters (*A*) is not straightforward. Still, linear differential equation models have been fit efficiently to expression data by various means, most prominently by recasting the problem into other more convenient forms [9,19,20]. When **f** is nonlinear, as is typically the case when trying to make more detailed models of network dynamics, then solving the minimization (Eq. 2) is all the more difficult.

There is another major approach to fitting ODE models, however, via functional data analysis (FDA) [21]. The fundamental idea of FDA is to transform a data series (e.g., the time series **y**(*t_i_*)) into a continuous function (**ŷ**(*t*)). This transformation often involves "denoising" the data, using smoothing splines or some other basis function approximation. Various estimation problems can then be solved in terms of these functions. FDA approaches have made some inroads in the literature on gene expression analysis. It has particularly appeared in papers on dimensionality reduction, clustering or classification for microarray expression time-series data (e.g., [22-24]). More relevantly to the present paper, several works have proposed estimating linear dynamical models from (microarray) expression data [25,26]. As will be explained in greater detail below, this approach to dynamical modeling allows the estimation problem to be reduced to one of regression, which carries both statistical and computational advantages.

There are several approaches to using FDA ideas in estimating differential equations [21,27]. For the general problem of estimating differential equation parameters with nonlinear (or linear) dynamics function **f**, the most direct approach is to create the smooth of the expression series **ŷ**(*t*) and then to differentiate that to produce . The model parameters can then be fit so that they recreate the estimated derivates as accurately as possible, rather than recreating the observed trajectory as accurately as possible.(3)

This error criterion is different from Equation 2. We will call that one trajectory-based error, and Equation 3 the derivated-based error. The FDA approach thus changes the problem being solved, rather than being an alternative method for solving the traditional formulation of ODE fitting. The derivative-based error has several major computational advantages that allow it to be optimized much more efficiently. First, evaluating the derivatived-based error for any particular parameter set *θ* is more efficient than for trajectory-based error. It does not require computation of a solution to the ODE (Eq. 1), but only evaluating the dynamics function **f** along the estimated trajectory **ŷ**(*t*). Depending on how **ŷ**(*t*) is represented, its derivative, , may be efficiently calculable as well. A second major advantage is that for typical models, the parameters *θ* can be partitioned into subsets *θ_g_* that are specific to each gene *g*'s dynamics. In this case, the derivative-based error decomposes into a sum of terms for each gene.(4)

Here,  denotes the element of the vector  corresponding to gene *g,* and similarly *f_g_* denotes the element of **f** pertaining to gene *g*. Thus, if there are *N* genes and, say, *K* free parameters per gene, the fitting problem is reduced from a single nonlinear *N* × *K*-dimensional problem to a set of *N* independent *K*-dimensional optimization problems. Such a reduction in dimensionality is typically very favorable when solving nonlinear optimization problems. Finally, although the derivative-based error criterion is still a nonlinear optimization problem for arbitrary dynamics functions **f**, informally, the optimization tends to be "less nonlinear" than for the trajectory-based error. In part, this is because the error involves only the evaluation of the dynamics function rather than solutions to the dynamics equation. Typically, **f** is not taken to be anything more complicated than a generalized linear model [28], so that minimizing derivated-based error is a generalized-linear least squares regression problem–a type of problem routinely solved in statistical analyses.

Despite the potential advantages of the FDA approach, we believe it has not been seriously evaluated on the problem of estimating nonlinear models of gene expression dynamics. In particular, neither its efficiency nor its ability to correctly estimate regulatory network architecture have been evaluated. Here, we formulate and test FDA approaches on data from two different real networks, the gap gene system of *Drosophila* melanogaster [7,8] and the synthetic IRMA network [29], and on simulated data generated by the GeneNetWeaver program [30]. We show that the FDA approach is extremely efficient at fitting nonlinear dynamical models of these data sets. In fact, it is so efficient that we can explicitly enumerate and test all possible regulatory architectures, which, to our knowledge, has never been achievable before for this type of modeling. These enumerations clarify the key regulatory factors, as well as interactions between factors, that explain the observed expression dynamics. We also assess the accuracy with which regulatory relationships are correctly extracted from the data, and compare it to other state-of-the-art fitting approaches. In general, the approach seems as successful as any other at determining which genes regulate which, and is very successful at discriminating the types of regulatory interactions–activation or repression.

## Results and discussion

### Systems and data

We apply FDA methods for fitting differential equation models of data from two real gene networks and simulated data from a set of *in silico* systems. Here we briefly describe these systems and the expression data upon which our fits are based.

#### The trunk gap gene system of Drosophila melanogaster

**Figure 1:**
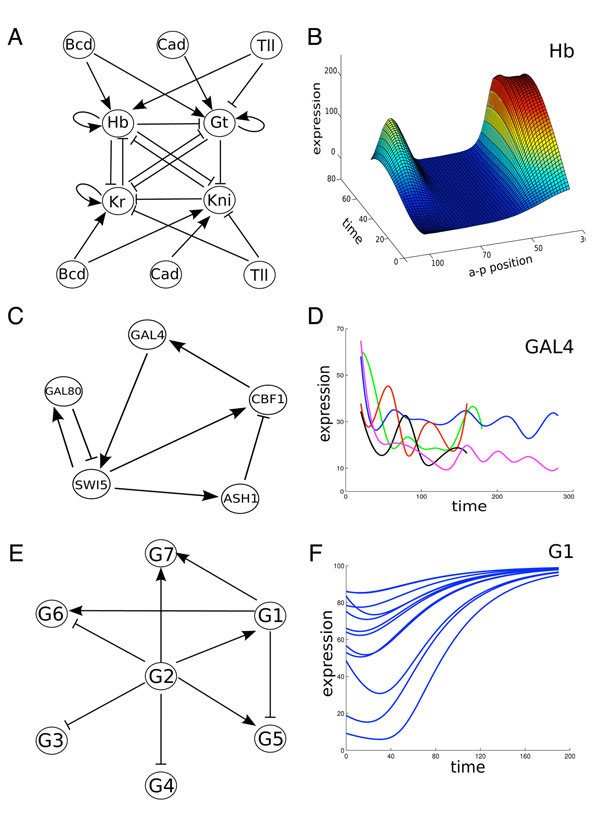
Networks and data used in our computational experiments. (A) A consensus model of regulatory interactions in the gap gene system of *Drosophila.* (B) Protein expression data for the Hb gene, as a function of anterior-posterior position along the embryo's trunk (measured in *%* embryo length) and time (minutes, starting at cleavage cycle 13). (C) Regulatory interactions in the synthetic IRMA network. (D) Five different expression trajectories for GAL4 mRNA in the IRMA network. (E) An example *in silico* network constructed by the GeneNetWeaver program. (F) Sample expression time series for gene G1 in that network.

The trunk gap gene system in *Drosophila* is part of the segmentation network, which is responsible for establishing patterns of gene activity early in the development of the embryo. These patterns mark off different regions, or segments, along the anterior-posterior axis of the embryo. There are four trunk gap genes: hunchback (Hb), Krüppel (Kr), giant (Gt) and knirps (Kni). It is known from extensive genetic studies that their activities are due to regulation amongst themselves, as well as input from at least three other genes: bicoid (Bcd), caudal (Cad) and tailless (Tll). While there remain some disagreements about details of these regulatory relationships, a broad consensus network model is presented in Figure 1A. All regulatory interactions between the trunk gap genes are repressive, while the factors Bcd and Cad activate different sets of genes. Tll activates Hb but represses the other trunk gap genes.

Reinitz and colleagues have made detailed measurements of the protein expression of these seven genes during development of the embryo [31] by confocal imaging of fluorescent antibody-labelled preparations. We use a data set that includes measurements at 8 different times spanning approximately one hour of actual time, and covering the trunk region of the embryo at a resolution of 1% of embryo length. This includes 7 genes × 8 times × 58 space points = 3248 total expression measurements. The data for the Hb gene are shown in Figure 1B.

#### A synthetic gene network in yeast

Cantone *et al.*[29] reported on the construction and mRNA expression measurement of a synthetic gene network created in yeast called IRMA (for *in vivo* benchmarking of reverse-engineering and modeling approaches). The network was constructed from five genes: SWI5, CBF1, GAL4, GAL80 and ASH1. Each gene was given a known and well-characterized promoter responsive to one or more of the other genes in the network, as shown in Figure 1C. In their paper, Cantone *et al.* describe GAL80 as repressing GAL4, but this is via their natural protein interaction properties. At the mRNA level, GAL80 does not affect GAL4, and so the effect of GAL80 is seen only at the target of GAL4 protein, which is the promoter of SWI5. Hence, our canonical model has GAL80 repressing SWI5. The endogenous transcription factors were deleted from the organism, to limit the impact of external factors. The dependence of GAL80/GAL4 binding on galactose is used as an on/off switch for the network. In the presence of galactose, the SWI5 gene is activated to subsequently trigger expression in the rest of the network. We use five different time-series that they generated by switching the network on using galactose and measuring the mRNA levels every 20 min over a five hour interval by quantitative real-time RT-PCR (see Figure 1D).

#### Test problems generated by GeneNetWeaver

GeneNetWeaver [30] can be used to generate *in silico* datasets of the expression dynamics of gene networks, and has been the basis for part of the DREAM network reverse engineering challenge for several years running [32]. The tool allows one to generate data from estimated yeast or *E. coli* networks, or subnetworks thereof. The program generates a kinetic model of gene expression, and can output time-series or steady state data for wild-type and genetic perturbation conditions. We used GeneNetWeaver to generate four *in silico* networks, two of which are sparsely connected like the IRMA network, which we denote S1 and S2 (see Figure 1E for an example), and two of which are more densely connected like the gap gene network, which we denote D1 and D2. We generated 20 wild-type expression time series for each network as the basis for model estimation (see Figure 1F).

### Unconstrained model-fitting by FDA

We smoothed and transformed the time series into continuous functions of time using the cubic spline functions built into the Matlab programming language (see Methods for details). For each of the data sets, this results in a set of functions **ŷ***^i^*(*t*), where the superscript *i* indicates it is the *i^th^* such time series–one of 58 for the *Drosophila* data, corresponding to each space point, one of 5 for the Cantone data, and one of 20 for each GeneNet Weaver network. With the cubic spline representation, the temporal derivatives can be directly obtained from the spline coefficients, so that  is readily computed.

We modeled the gene expression dynamics by differential equations of the form(5)

where *R_g_* is the maximum rate of production of gene *g*'s protein or mRNA,  is a sigmoidal function ranging between zero and one, *T_gg_*′ is the regulatory weight describing the effect of gene *g*′ on the production of *g*'s protein or mRNA, *h_g_* is a bias term, and *λ*_g_ is the decay rate.

For each gene *g,* fitting such an equation to the smoothed data by the FDA approach amounts to finding parameters that minimize the error function(6)

where *i* ranges over the trajectories in the data set and *t* is integrated over the duration of the trajectories. This optimization problem was sufficiently tractable to be solved by the simplex-based search procedure fmincon in Matlab, using repeated runs from different initial conditions, as described in the Methods section. This error function contains no regularization to encourage "simple" explanations of the data and/or to prevent overfitting. So, we also tried adding an *L*_1_ penalty to the error function(7)

where *E*_0_ is the original error function of Equation 6 and *c* is a parameter determining the relative import of fitting the data accurately and using "small" weights. The *L*_1_ penalty is often used in an attempt to eliminate excess parameters in regression problems. If one is only concerned about prediction accuracy, and if one has statistically independent data points, then cross-validation can be used to choose a value of c that appropriately trades off model complexity and model accuracy on the training data. In our case, the data come from time series, so derivative estimates at different times are certainly not statistically independent. Nor is our primary concern the accuracy of the regression model. This is only a conduit to determining regulatory architecture. Thus, we experimented with a range of c values, as described in more detail below. Regulatory weights that remain nonzero for large values of *c* are the most important for explaining derivatives, and we give these the highest "confidence".

For the IRMA and GeneNetWeaver data sets, we fit models without autoregulatory links, as these systems do not include autoregulation. For the gap gene system, however, where autoregulation is believed to occur, we allowed autoregulatory links in the model. Three of the *Drosophila* genes and some of the genes in the GeneNet Weaver networks do not have any regulatory inputs–at least, not among the genes considered. We did not model these genes, restricting our modeling efforts (and accuracy assessments) to those genes that are regulated.

#### Results on the Drosophila data

**Figure 2:**
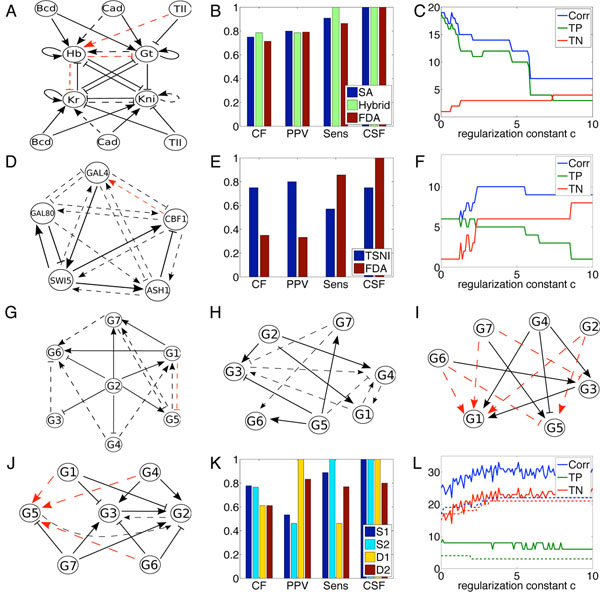
Results of unregularized and *L*_1_-regularized fitting without constraint on regulatory architecture. (A) The estimated regulatory architecture for the gap gene network. Dashed black links are false positives that are not in the gold standard model. Dashed red are missing links that are in the gold standard. (B) Statistics regarding the accuracy of regulatory architecture, as estimated by the simulated annealing (SA) approach of Jaeger *et al.*[7,8], the hybrid optimization approach of Perkins *et al.*[33], and the functional data analysis (FDA) approach tested in this work. CF is the fraction of relationships correctly identified as –, 0 or +; PPV is positive predictive value; Sens is sensitivity; and CSF is the fraction of nonzero links in both the gold standard and the estimated architecture that have the same sign (+ or –). (C) The effects of *L*_1_-regularization on total correct links (Corr), true positives (TP; interpreted as links shared by the gold standard and the model, regardless of sign) and true negatives (TN; interpreted as links absent in both the gold standard and the model). (D-F) The same information for the fits to the IRMA data. In panel E, TSNI refers to the best-performing approach as tested by Cantone *et al.*[29]. (G-J) Estimated architectures for the GeneNet Weaver networks S1, S2, D1 and D2 respectively. (K) Summary statistics for the accuracy of FDA reconstruction of the regulatory archictectures. (L) The *L*_1_-regularized performance of the FDA approach on the sparse networks S1 (solid lines) and S2 (dashed lines).

Figure 2A shows the network architecture estimated by minimizing the *E*_0_ error. Only three true links are missed: repression of Kr and Gt by Hb, the latter of which is a comparatively weak effect [8], and activation of Hb by Tll (for which the weight was just below threshold). The model posits five false positive links that are not part of the gold standard (Figure 1A). Figure 2B gives some statistics summarizing the accuracy of the reconstructed regulatory architecture, and comparing to two previous fits obtained using different methods on the same data. Jaeger *et al.*[7,8] used a long-running simulated annealing (SA) method to optimize a trajectory-based notion of goodness-of-fit, whereas Perkins *et al.*[33] used a hybrid approach that combined derivative estimation and fitting, similar to functional data analysis, with trajectory-based optimization. Both previous approaches were considered to generate highly accurate fits, both in terms of squared error and estimated regulatory architecture. Panel B shows that all three approaches get very similar numbers of links correct, where correctness is interpreted as matching the gold standard in terms of negative interaction, no interaction, or positive interaction. All three approaches also have very similar positive predictive value and sensitivity. All methods performed perfectly on what we call the correct sign fraction (CSF), which is the fraction of nonzero links in both the gold standard and estimated models that have matching sign. In other words, none of the links in the models produced by these methods are activating when the real relationship is repressing, or vice versa. Thus, all three fitting approaches appear to have very similar success in extracting regulatory relationships from the data. The FDA approach achieves this performance with drastically improved computational efficiency compared to either of the other approaches. The simulated annealing approach used by Jaeger *et al.* took on the order of months of computation time for their complete study, while the approach of Perkins *et al.* took on the order of tens of hours. The FDA fitting takes tens of minutes. Although general improvements in computing speed may be the cause of some of this improvement, the FDA approach is clearly faster than previous approaches to nonlinear model fitting. Pilot tests suggest that it could readily be sped up by another factor of 10 or so simply by using fewer repeats of the search procedure (see Methods) with little loss in quality.

The gap gene network is densely connected, with 22 of the 28 possible links present in the gold standard model. Adding regularization to the optimization criterion risks eliminating true positives. Nevertheless, we tried optimizing the *L*_1_-regularized error function *E*_1_ for regularization constant c ranging from 0 to 10 in increments of 0.1. The results are summarized in Figure 2C. As one would hope, increasing c increases the number of true negatives from 1 (at *c* = 0) to 4 (at *c* = 10). At the same time, however, the number of true positives, and total correct links, drops drastically. The positive predictive value does not improve with *c*, as both true positives as well as false positives are dropped from the model (data not shown).

#### Results on the IRMA data

Figure 2D shows the network obtained by optimization of the *E*_0_ criterion for the IRMA data. The IRMA network is sparse compared to the gap gene network, having only seven links among the five genes. The optimization correctly identifies six of those links, including their correct sign. It misses only the activation of GAL4 by CBF1, perhaps because the model also has the true regulators of CBF1 connected to GAL4-a case of mistaking direct versus indirect regulation. Without regularization, however, there are many false positive links in the estimated regulatory architecture. Figure 2E compares the performance of the *E*_0_ optimization against the TSNI algorithm, which fits a linear differential equation model that is limited to at most two inputs per gene. This algorithm performed the best of several alternatives tested by Cantone *et al.*[29]. The TSNI algorithm detected four of the seven true links in the network, attributing the correct sign to three of those links. Its overall fraction of correct links is much higher than that for the FDA fit, perhaps in part because the limitation to two inputs per gene ensures many true negatives–links absent in both the gold standard and the estimated model. (In the next section, we will see how FDA performs when limited to two inputs per gene.) Because of the large number of false positives in the FDA fit, it also has significantly lower PPV than TSNI. However, the FDA fit enjoys greater sensitivity and correct sign fraction.

Because the unregularized fit includes a large number of false positives, we hoped that adding the *L*_1_-regularization would improve the accuracy of the estimated network architecture. Figure 2F shows the results for regularization constant *c* ranging between 0 and 10. Regularization was partly successful. For c ranging from roughly 3 to 5, one of the true positives was lost, but five false positives were also trimmed away, approximately halving their number. This still left seven false positives, however, which could only be eliminated by losing most of the true positives. From both our experience and the results of Cantone *et al.*[29], the IRMA dataset appears much more challenging than the gap gene data, perhaps due to the much smaller number of time series (only 5, compared to 58) or to noise in the data (the gap gene data incorporates significant smoothing and averaging across embryos, to eliminate observation noise and other sources of variability).

#### Results on the GeneNetWeaver data

Broadly speaking, our results on the two sparse GeneNetWeaver networks mimicked our results on IRMA, and our results on the two dense GeneNetWeaver networks mimicked our results on the gap gene network. Figure 2G-J show the estimated network structures. For the sparse networks all (S2) or nearly all (S1) true links are detected and all are correctly signed. However, there are significant numbers of false positives–albeit less than in the IRMA fit. Conversely, the estimates for the dense networks include no (D1) or few (D2) false positives, but miss out identifying some true links. One particularly interesting case is gene G1 in network D1. This gene has five regulators, all of which act positively, and only two of which are identified by the FDA fit. The other regulators are difficult to detect because the gene is nearly always being activated, and so intuitively it appears almost as if it is unregulated–it is rare to observe the gene in a state that reveals anything about its regulation. The basic trends in true and false positives are reflected in the summary statistics shown in Figure 2K. PPV is moderate for the sparse network and high for the dense networks, while sensitivity is the opposite. One difference in comparision with the gap gene and IRMA results is that FDA obtains higher fractions of correctly signed links (activation, repression or no effect) on the sparse networks than on the dense networks. In all cases performance is significantly better than chance, which would only be right 1/3 of the time.

For the two sparse networks, where false positives were a concern, we evaluated the *L*_1_-regularization approach to improving accuracy. The results are shown in Figure 2L. For both networks, regularization was able to eliminate the majority of the false positives, with little loss in true positives. For S1, the number of correct links (Corr) reached as high as 33 out of 36 links for regularization constant c around 4 or 5. For S2, the number of correct links was as high as 24 out of 30.

### Explicit enumeration of possible network structures

As mentioned above, the FDA approach to model fitting is computationally efficient. Part of its speed is due simply to the greater ease of evaluating the derivative-based error (Eq. 3) as opposed to the more traditional trajectory-based error (Eq. 2). We tested this in Matlab, comparing our implementation of the derivative-based error against a trajectory-based error function that uses the built-in ode45 function to solve the dynamics equation. Over a range of testing conditions, we found that the derivative-based error could be computed 300 ±40 times faster than trajectory-based error.

One of the advantages of the speed with which the FDA fits can be done is that we do not need to limit ourselves to unconstrained network architectures. We can explicitly test alternative architectures and, in fact, we are able to enumerate them all if the number of genes in the network is not too large. For the gap gene network, where all seven of the measured genes can act as input to any of the gap genes, there are 2^7^ = 128 possible input combinations for any gene. Because each gene's model is fit independently, we can test all possible regulatory architectures with a total of 4 × 2^7^ = 512 fits. This begins to be a significant computation, but on a 32-core computing cluster, it amounted to an overnight job. By enumerating all possible inputs for every gene, we are able to explicitly assess which regulators or combinations of regulators are most important for explaining each gene's observed expression. Enumeration also gives us another way to regularize the fit, by limiting the number of inputs per gene.

**Figure 3:**
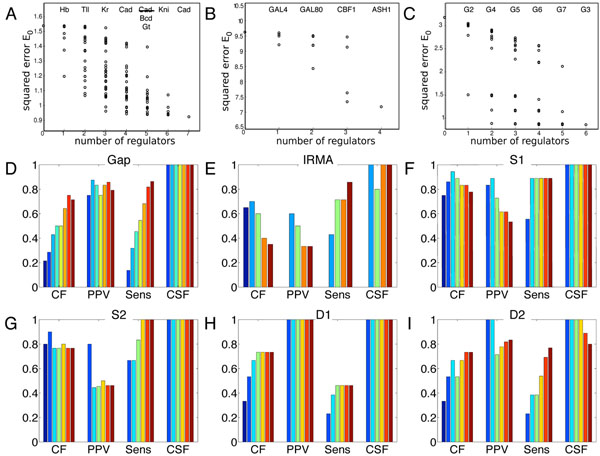
Results of enumerating all possible regulatory architectures. (A) For Hb in the gap gene network, the *E*_0_ error of each possible input set is plotted on the y-axis, with the size of the input set on the x-axis. (B) A similar plot for SWI5 in the IRMA network, and (C) for gene G1 in the sparse network S1 generated by the GeneNetWeaver software. (D-I) Statistics on regulatory network accuracy using the best input combination of each size (colors dark blue through dark red indicate zero inputs through all possible inputs). For definitions of CF, PPV, Sens and CSF, see text or Figure 2 caption.

We performed enumerations for all six networks. The results are summarized in Figure 3. Panels A through C show the scores of all possible input combinations for several example genes. Panel A, for instance, shows the scores of the different input combinations in explaining the Hb gene in the gap gene network. At the top of each column of points, we list the regulator whose addition constitutes the lowest-error input set for the given number of regulatory inputs. For example, the single best factor for explaining Hb's dynamics is Hb itself–which is correct, as Hb autoactivation is well established [8]. If two regulators are allowed, then the best combination is Hb and Tll, that latter of which helps to activate the posterior Hb expression domain. With three regulators, repression from Kr is added to the mix, by which point most of the variability in the data that can be explained by the model is explained. The fourth regulator to be added is Cad, which is incorrect, but barely improves quality of fit. If five regulators are allowed, then the optimal combination does not include Cad, but rather two other factors, Bcd and Gt. We found that in our gap gene fits, the single best regulator was always the gene itself. In some cases, this may be right. But in other cases is it likely wrong, and arises from confusing correlation with causation: expression of a gene requires regulatory activation, thus expression indicates activation, even though it does not cause it. This phenomenon did not occur in the IRMA and GeneNetWeaver fits, as autoregulation was disallowed in those cases. Figure 3B shows the scores of different input combinations for the SWI5 gene in the IRMA network. The first two regulators identified, GAL4 and GAL80 are the true and only regulators. However, the plot shows that adding regulation by CBF1 to these two significantly reduces the error even further, even though it is not a true regulator. This kind of error profile was more characteristic of the IRMA genes (data not shown). Figure 3C shows the errors of input combinations for the G1 gene in the S1 network, which was typical for the GeneNetWeaver networks. Usually, the first one or two regulators explained nearly all of the explicable variability in the data–in this case, with one true positive link and one false positive link.

Figure 3D-I give summary statistics for the accuracy of the regulatory networks when limited to the best single-input, two-input, etc. models of each gene. By necessity, the fraction of correct links (CF) is low for the dense networks when only one or a few inputs are allowed, but increases as more inputs are allowed. The reverse happened for the IRMA network, where limiting to one or two inputs gave much better CF and PPV scores, though still not quite as high as achieved by TSNI. Interestingly, for the sparse networks S1 and S2, CF did not vary significantly with the number of regulators allowed. When extra regulators were allowed, the optimization could often drive superfluous weights to near zero, so that they fell below our significance threshold and were counted as zero links.

**Figure 4:**
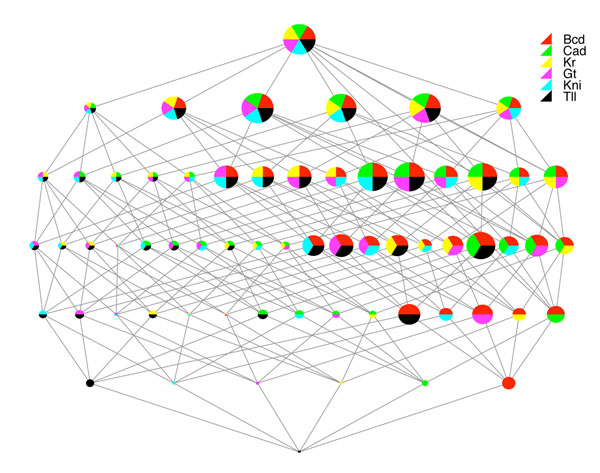
A visual depiction of the scores of different input combinations for the Hb gene in the gap gene network, omitting autoregulation. The graph structure depicts the partial ordering of all possible input combinations, with the no-inputs case at the bottom and all possible inputs at the top. The colors within the circles indicate the genes participating in the combination, as laid out in the key at the upper right. The size of each circle is inversely related to the error obtained by using that combination, so that small circles indicate high error and large circles indicate the smallest error.

Figure 4 shows another way of displaying the relative errors of different combinations of regulatory inputs. In this case, the plot is for Hb, and we consider only combinations without Hb autoregulation. The chart shows that, without Hb as an option, the single best regulator for Hb is Bcd (the red circle), which is known to be the primary activator of Hb's primary expression domain towards the anterior of the trunk. The second best single regulator is Tll, which, as mentioned above, is responsible for Hb's secondary, posterior expression domain. The best two-regulator combination is Bcd with Tll, though Bcd with Gt is nearly as good, and so on. In fact, Bcd is an important regulator nearly regardless of what other regulators are present in the model, as witnessed by the fact that all the circles lacking Bcd (red) are much smaller than the circles with a Bcd component. While it is beyond the scope of this paper to go through detailed analyses of different genes, we believe that this sort of combinatorial analysis and display is extremely useful in assessing the relative importance of different candidate regulatory inputs and their combinations. Thus, we conclude that the speed and accuracy of model fitting by FDA, and the subsequent analyses it enables, strongly recommend it as a tool for genetic network inference.

## Conclusions

Our computational studies show that functional data analysis is a powerful approach to estimating nonlinear models of gene expression dynamics, and in particular, to estimating the regulatory relationships between genes. The accuracy of FDA was comparable to state of the art approaches on both the gap gene [7,8] and IRMA [29] data sets, which have previously been analyzed by a number of methods, and it performed well on synthetic data sets generated by the GeneNetWeaver program [30]. The FDA approach is computationally efficient because it transforms the estimation problem into a decomposable multiple regression problem. This efficiency enables in-depth analysis of the influences of different factors, as well as explicit exploration of all possible regulatory input combinations.

As with any estimation problem, overfitting-avoidance is an important consideration. We explored *L*_1_-regularization as well as explicitly limiting the number of regulators allowed for each gene.

*L*_1_-regularization was partly successful on the sparse IRMA network, and much more successful on the sparse GeneNetWeaver networks. *L*_1_-regularization requires a constant, *c*, which determines the relative importance of accuracy of fit to the data versus model complexity (in the sense of summed absolute values of the regulatory weights). Testing a range of values for *c* allows us to identify links that are most important for accounting for the data. Large *c* means regulatory weights are highly penalized. Weights that remain significantly different from zero at large *c* are the best predictors, and thus represent the links in which we have the most confidence. Although there is no standard procedure for choosing a "best" value of *c* for this sort of data and fitting problem, empirically a value around *c* = 4 or *c* = 5 resulted in the highest accuracy of network reconstruction. That a common value worked for all networks is, no doubt, partly due to the similar scales of the expression data (after normalization) and the similar numbers of candidate regulators per gene. Still, it is surprising that common values of c emerged despite quite different numbers of time series for each network and different densities of regulatory links in the networks.

Explicitly evaluating all possible combinations of regulators allows one to see which combinations are the best predictors. In particular, this allows one to identify the best 1-input model of each gene, the best 2-input model, and so on. So, it provides another means for determining which candidate regulators are most important. At the same time, it reveals whether there are alternative solutions of nearly equal quality, and generally gives a more in depth view of the contributions of different regulators, especially when used in conjunction with visualizations methods, as shown in the Results section.

The approach that we have described for using FDA to estimate nonlinear differential equation models of gene expression dynamics can be extended in various ways. One important extension would be to accommodate genetic perturbation data, such as knock-outs, knock-downs or overexpression conditions. In the case of a complete knock-out, this is readily handled by hard-wiring expression of the knocked-out gene to zero in the model and otherwise fitting the data as usual. However, for partial knockdowns or overexpressions of unknown or time-varying magnitudes, more sophisticated procedures are needed. Another relevant extension would be to allow for delays in the differential equations. Cantone *et al.,* for example, suggest that delays may be relevant to modeling their system [29]. For known delays, the FDA framework extends trivially to accommodate delay differential equations, but when delays are unknown, more elaborate extensions are needed [21]. Finally, another natural generalization to explore would be to Bayesian parameter estimation frameworks. Because FDA reduces the parameter estimation problem to one of nonlinear regression, standard methods for approximate computation of posteriors over parameters in nonlinear regression could be applied [34]. Alternatively, if one is interested in Markov-chain Monte Carlo [35] or reversible-jump Markov-chain Monte Carlo [36] approaches to Bayesian parameter and/or network structure estimation for genetic networks, then the efficiency of evaluating the data likelihood under an FDA model, and the decomposition of the problem into separate genes, should be of great advantage.

## Methods

### Data smoothing

To obtain the temporal derivatives of the time series data, it is necessary to obtain a functional representation of the data. We constructed continuous-time series by interpolating the data with cubic splines, as implemented in the Matlab Spline Toolbox. This toolbox also includes a function to compute the derivatives from the spline. Cubic splines are not wholly defined by the data, but also depend on assumptions at or near the boundaries–in our case, the start of the time series and the end of the time series. The default approach taken by the Matlab's spline function is to use the "not-a-knot" assumption, which states that the third derivative of the spline function should be continuous at the second knot point and the next-to-last knot point [37]. Matlab offers other approaches for completing cubic splines. In pilot studies, we tried the default (not-a-knot) approach, natural cubic splines (which have second derivatives equal to zero at the endpoints; Matlab calls this the "variational" approach), and Matlab's "complete" approach (which sets first derivatives at the endpoints based on an estimate from the function values at the nearest four knots). We found that these different methods for completing the cubic splines had only small effects on the interpolated curves and negligible effects on parameter estimates for our models. So, throughout this paper we used the default not-a-knot approach.

For the IRMA and GeneNetWeaver data sets, we also experimented with smoothing the data first, using the smooth function of Matlab, but this did not affect results significantly. For the *Drosophila* data, we smoothed/simplified the data by eliminating certain time and space points before interpolating with cubic splines. The space and time points that we eliminated were selected by an evolutionary strategy that sought to minimize a criterion that combined the squared error between the original data and the cubic-splined interpolation at the same point and a penalty for small fluctuations in the derivative.

### Fitting details

Minimization of the *E*_0_ or *E*_1_ criteria was done by the Matlab function fmincon. For each optimization, we did 1000 runs from different randomized starting conditions, initializing parameters uniformly within their allowed intervals. For *Drosophila* the expression data ranged between 0 and 255, regulatory weights were constrained to [–0.1, 0.1], production rates to [0, 25], and decay rates to [0,10]. The bias term was fixed at –3.5, following previous work [8]. For the IRMA and GeneNetWeaver networks, the expression data was multiplied by 100, so that it fell in the range [0, 100]. Weights were constrained to [–0.2,0.2], production terms to [0, 25], decay terms to [0,10] and bias terms to [–25, 25]. Of the 1000 runs, the one resulting in the lowest error was reported as the solution. (Typically many runs found solutions with nearly the same weights and nearly the same error. We never observed two clearly distinct solutions of equal or near equal quality.) Weights less than 0.006 in magnitude were considered zero, and were otherwise considered positive or negative, depending on their sign. For the enumerations, we used the same fitting procedure except that links that were not part of the optimization were contrained to be zero.

### GeneNetWeaver

For the GeneNetWeaver [30] experiments, we used the program 30 times to generate networks of seven genes. From these, we chose the two sparse networks and the two dense networks to generate data. The data were produced according to the rules of the DREAM5 contest, except without noise. Numerous papers have addressed the removal of noise from this data as part of inference (e.g., [32,38]). The program generates time series by applying a perturbation from the steady state for a period of time *T,* and then removing the perturbation and letting the network relax back towards the steady state for an equal period of time. We used the second half of the time series, as it describes the wild-type (unperturbed) behavior of the network.

## List of abbreviations used

ODE = Ordinary differential equation

FDA = Functional data analysis

CSF = Correct sign fraction

PPV = Positive predictive value

IRMA = A synthetic gene network created in yeast, and reported by Cantone *et al.*[29]

TSNI = A fitting algorithm identified as the best-performing among several alternatives investigated by Cantone *et al.*[29] on the IRMA data

DREAM = An annual contest on reverse engineering

## Competing interests

None.

## Authors contributions

GS and TJP conceived the experiments, analyzed the data, and wrote the paper. GS conducted the computational experiments.

## References

[B1] Yuh CH Bolouri H Davidson EH Genomic cis-regulatory logic: experimental and computational analysis of a sea urchin gene Science 1998 279 1896 1902 10.1126/science.279.5358.1896 9506933

[B2] Von Dassow G Meir E Munro EM Odell GM The segment polarity network is a robust developmental module Nature 2000 406 188 192 10.1038/35018085 10910359

[B3] Albert R Othmer HG The topology of the regulatory interactions predicts the expression pattern of the segment polarity genes in *Drosophila melanogaster* Journal of Theoretical Biology 2003 223 1 18 10.1016/S0022-5193(03)00035-3 12782112 PMC6388622

[B4] Ma W Lai L Ouyang Q Tang C Robustness and modular design of the Drosophila segment polarity network Molecular Systems Biology 2006 2 10.1038/msb4100111 17170765 PMC1762089

[B5] Li F Long T Lu Y Ouyang Q Tang C The yeast cell-cycle network is robustly designed Proc 2004 101 14 4781 10.1073/pnas.0305937101 15037758 PMC387325

[B6] Fauré A Thieffry D Logical modelling of cell cycle control in eukaryotes: a comparative study Mol 2009 5 1569 1581 10.1039/b907562n 19763341

[B7] Jaeger J Surkova S Blagov M Janssens H Kosman D Kozlov KN Manu Myasnikova E Vanario-Alonso CE Samsonova M Sharp DH Reinitz J Dynamic control of positional information in the early *Drosophila* embryo Nature 2004 430 368 371 10.1038/nature02678 15254541

[B8] Jaeger J Blagov M Kosman D Kozlov KN Manu Myasnikova E Surkova S Vanario-Alonso CE Samsonova M Sharp DH Reinitz J Dynamical analysis of regulatory interactions in the gap gene system of *Drosophila melanogaster* Genetics 2004 167 1721 1737 10.1534/genetics.104.027334 15342511 PMC1471003

[B9] D'Haeseleer P Wen X Fuhrman S Somogyi R Linear Modeling of mRNA Expression Levels During CNS Development and Injury In Proceedings of the Pacific Symposium on Biocomputing 1999 41 52 10.1142/9789814447300_0005 10380184

[B10] De Hoon M Imoto S Kobayashi K Ogasawara N Miyano S Inferring gene regulatory networks from time-ordered gene expression data of Bacillus subtilis using differential equations Pacific Symposium on Biocomputing 2003: Kauai, Hawaii, 3-7 January 2003 2002 World Scientific Pub Co Inc 17 full_text 12603014

[B11] Janssens H Hou S Jaeger J Kim A Myasnikova E Sharp D Reinitz J Quantitative and predictive model of transcriptional control of the *Drosophila melanogaster even skipped* gene Nature Genetics 2006 36 1159 1165 10.1038/ng1886 16980977

[B12] Jaeger J Sharp DH Reinitz J Known maternal gradients are not sufficient for the establishment of gap domains in *Drosophila melanogaster* Mechanisms of Development 2007 124 108 128 10.1016/j.mod.2006.11.001 17196796 PMC1992814

[B13] Nahmad M Glass L Abouheif E The dynamics of developmental system drift in the gene network underlying wing polyphenism in ants: a mathematical model Evolution & Development 2008 10 3 360 374 18460097 10.1111/j.1525-142X.2008.00244.x

[B14] Mendoza L Alvarez-Buylla ER Dynamics of the Genetic Regulatory Network for *Arabidopsis thaliana* Flower Morphogenesis Journal of Theoretical Biology 1998 193 307 319 10.1006/jtbi.1998.0701 9714934

[B15] Huang S Eichler G Bar-Yam Y Ingber D Cell fates as high-dimensional attractor states of a complex gene regulatory network Physical review letters 2005 94 12 128701 10.1103/PhysRevLett.94.128701 15903968

[B16] Huang S Guo Y May G Enver T Bifurcation dynamics in lineage-commitment in bipotent progenitor cells Developmental Biology 2007 305 2 695 713 10.1016/j.ydbio.2007.02.036 17412320

[B17] Akutsu T Kuhara S Maruyama O Miyano S Identification of Gene Regulatory Networks by Strategic Gene Disruptions and Gene Overexpressions In Proceedings of the Ninth A CM-SIAM Symposium on Discrete Algorithms 1998 695 702

[B18] Avery L Wasserman S Ordering gene function: the interpretation of epistasis in regulatory hierarchies Trends in Genetics 1992 8 9 312 316 10.1016/0168-9525(92)90263-4 1365397 PMC3955268

[B19] Yeung MKS Tegner J Collins JJ Reverse engineering gene networks using singular value decomposition and robust regression Proceedings of the National Academy of Sciences of the USA 2002 99 6163 6168 10.1073/pnas.092576199 11983907 PMC122920

[B20] Bansal M di Bernardo D Inference of gene networks from temporal gene expression profiles IET Syst. Biol 2007 1 5 306 312 10.1049/iet-syb:20060079 17907680

[B21] Ramsay JO Silverman BW Functional Data Analysis 1997 Springer

[B22] Yao F Lee T Penalized spline models for functional principal component analysis Royal Statistical Society Series B Statistical Methodology 2006 68 3 10.1111/j.1467-9868.2005.00530.x

[B23] Leng X Muller H Classification using functional data analysis for temporal gene expression data Bioinformatics 2006 22 68 10.1093/bioinformatics/bti742 16257986

[B24] Song J Lee H Morris J Kang S Clustering of time-course gene expression data using functional data analysis Computational biology and chemistry 2007 31 4 265 274 10.1016/j.compbiolchem.2007.05.006 17631419 PMC1992527

[B25] Ando T Imoto S Miyano S Functional data analysis of the dynamics of gene regulatory networks 2005 Springer 69 8315658129

[B26] Opgen-Rhein R Strimmer K Inferring gene dependency networks from genomic longitudinal data: a functional data approach REVSTAT-Statistical Journal 2006 4 53 65

[B27] Ramsay J Hooker G Campbell D Cao J Parameter estimation for differential equations: a generalized smoothing approach Journal of the Royal Statistical Society-Series B 2007 69 5 741 796 10.1111/j.1467-9868.2007.00610.x

[B28] Hastie TJ Tibshirani RJ Generalized Additive Models 1990 Chapman & Hall/CRC

[B29] Cantone I Marucci L Iorio F Ricci MA Belcastro V Bansal M Santini S di Bernardo M di Bernardo D Cosma MP A Yeast Synthetic Network for In Vivo Assessment of Reverse-Engineering and Modeling Approaches Cell 2009 137 172 181 10.1016/j.cell.2009.01.055 19327819

[B30] Marbach D Schaffter T Mattiussi C Floreano D Generating realistic in silico gene networks for performance assessment of reverse engineering methods Journal of Computational Biology 2009 16 2 229 239 10.1089/cmb.2008.09TT 19183003

[B31] Poustelnikova E Pisarev A Blagov M Samsonova M Reinitz J A database for management of gene expression data in situ Bioinformatics 2004 20 2212 2221 10.1093/bioinformatics/bth222 15059825

[B32] Marbach D Prill R Schaffter T Mattiussi C Floreano D Stolovitzky G Revealing strengths and weaknesses of methods for gene network inference Proceedings of the National Academy of Sciences 2010 107 14 6286 10.1073/pnas.0913357107 PMC2851985 20308593

[B33] Perkins TJ Jaeger J Reinitz J Glass L Reverse Engineering the Gap Gene Network of *Drosophila melanogaster* PLoS Computational Biology 2006 2 5 e51 10.1371/journal.pcbi.0020051 16710449 PMC1463021

[B34] Bishop CM Pattern Recognition and Machine Learning 2007 Springer

[B35] Doucet A de Freitas N Gordon NJ Sequential Monte Carlo Methods in Practice 2001 Springer-Verlag

[B36] Green P Reversible jump Markov chain Monte Carlo computation and Bayesian model determination Biometrika 1998 82 711 732 10.1093/biomet/82.4.711

[B37] De Boor C A practical guide to splines 2001 Springer Verlag

[B38] Yip K Alexander R Yan K Gerstein M Improved reconstruction of in silico gene regulatory networks by integrating knockout and perturbation data PLoS ONE 2010 20126643 10.1371/journal.pone.0008121 PMC2811182

